# Alobar holoprosencephaly secondary to CMV infection

**DOI:** 10.11604/pamj.2014.17.8.2934

**Published:** 2014-01-14

**Authors:** Sanae Abakka, Mounia Yousfi

**Affiliations:** 1 Department of Obstetrics and Gynaecology, Oncology and High Risk Pregnancies, Maternity Hospital Souissi, Ibn Sina Teaching Hospital, Rabat, Morocco

**Keywords:** Alobar holoprosencephaly, CMV, infection

## Image in medicine

A 30 year old woman, gravida 3 para 2, was admitted to the labour ward at term. Routine ultrasound scan performed at 23 weeks had revealed a single cerebral ventricle, fused thalami and absent midline structures (Panel A), with anti-CMV IgG increasing from 250 to 539 IU/mL at 23 and 25 weeks. Antenatal diagnosis of alobar holoprosencephaly following congenital CMV infection was made. The parturient was lost to follow-up until term where she presented with a 3 cm dilated cervix. Ultrasound scan showed a biparietal diameter of 115 mm, leading to emergency cesarean section with extraction of a female infant, 3000 g, APGAR 1/1 not resuscitated, with cyclopia, synophtalmia and proboscis (Panel B). Alobar holoprosencephaly is a lethal malformation resulting from inadequate cleavage of the forebrain. The most common aetiologies are maternal diabetes, CMV, Rubella or toxoplasma infections, trisomy 13 and 18. Early termination of pregnancy should be offered.

**Figure 1:**
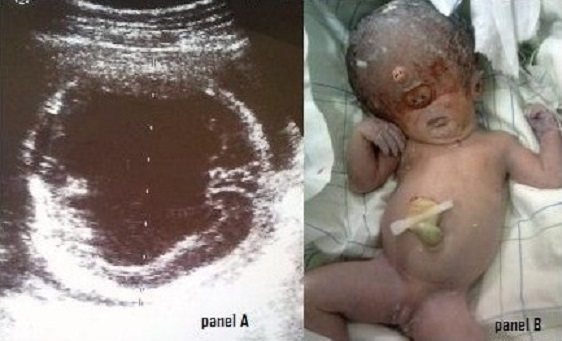
A) Ultrasound scan of the fetal head; B) Postnatal picture of the malformed infant

